# Communicability of Yellow Fever

**Published:** 1859-02

**Authors:** R. B. S. Hargis

**Affiliations:** U. S. Marine Hospital, Pensacola, Fla.


From the New Orleans Medical News.

*Communicability of Yellow Fever.* By R. B. S. HARGIS, M. D.

U. S. MARINE HOSPITAL, Pensacola, Fla., Nov. 3, 1858.

*Messrs. Editors:*—In the last (October) number of the New Orleans Medical News and Hospital Gazette, you quote some remarks from the St. Louis Medical Jour. "showing conclusively the non-contagiousness of yellow fever." I do not queston that statement, but will adduce another showing decisively that terrible malady *is* under *some* circumstances communicable from one person to another; and I shall not go beyond these walls for proofs.* I intend to be explicit but brief:

In September, 1854, Capt. Joseph Golder, of the bark "Alfred Exall," from Havana, was admitted into this institution with yellow fever. Having several cases of other diseases, and half a dozen or more convalescents from various affections, in accordance with a long established rule here, respecting contagious diseases, the patient was put into a separate ward, and all communication with him forbidden, except by his attendants. Being very sick, and consequently indifferent as to the rules, Golder allowed two of the convalescents to contravene them. Davidson, a convalescent, from a gastric disorder, wrote letters at his (Golder's) table, on the 20th of September; contracted the disease on the 23d; and died on the 24th, of black vomit. Bynton, recovering from a hæpatic affection did the same thing on the 23d of September, and succumbed on the 25th, in a puddle of the "fatal ejection." Each case exhibited, during its progress, all the characteristic phenomena of the most malignant type of yellow fever. Cadaveric autopsy confirmed the ante-mortem indications in both cases.

This year the hospital being crowded to such an extent as to prevent insulation of yellow fever cases, six convalescents, from other affections, contracted the disease.

John Rice, recovering from an injury, had not been in an affected port during the season, unadvisedly slept two or three nights next to a yellow fever case, sickened, had black vomit, and recovered.

---

*Pensacola has the deserved reputation of being the healthiest city on the Gulf coast. Her sanitary condition this season has been excellent. The Marine Hospital is situated about a mile from the suburbs of the city, in a beautiful open grove, on a bluff overlooking the bay; a dense forest intervenes between it and the city.

I make this statement to show that this hospital is "situated beyond any *infected district.*" Hence, in the instances of communicability of yellow fever mentioned in the text, *the morbific cause* MUST *have been reproduced.* To adduce further proofs is idle—"*On ne cherche point a prouver la lumiere.*"

George Way, three or four weeks from Galveston, was in the hospital a week with intermittent fever, got well, and was to be discharged on the morning of the 6th of October, but complaining of pain in the head and back, and a sense of weariness, was suffered to remain—he died on the 9th, with black vomit.

John Bell, Thomas Harper, John Hefferson, and George Curtis, all from healthy ports, contracted the yellow fever at different periods.—The first mentioned had had diarrhœa, the next intermittent fever, and the last a sore leg; he (the latter) died; the others recovered.

We have treated twenty-eight cases of yellow fever in this hospital since the 14th of September last; twenty-one recovered. Two of those who died were admitted *in articulo mortis*.

The following cases from the register will indicate in a measure the treatment, generally here, pursued:

*Case I.*—Charles Brown, aged 23 years, born in Boston; a robust fellow; admitted on the 14th of September, (1858), had been sick two days; had taken no medicine; complains of pain in the head, back and knees, and a sense of languor; has some nausea and thirst; is dizzy when sitting up; respiration prolonged; eyes injected; tongue furred; skin very warm; bowels confined. Let him have a hot mustard bath, semi-cupium; cups to the nucha; and an enema of sodii chloridum, and infus. lini., followed by pills of chlor. hydrarg. mitis ext. colocynth. co., and ext. hyosciam; also, infus. flor. sambuc. to be drank occasionally.

*September* 15*th*.—Has better appearance; skin a little moist; some headache and vertigo; bowels moved two or three times during last night. Gave him immediately sulph. quinine, grs. xx.; and ordered the dose repeated in two hours. Continue infus. flor. sambuc.

*Same day*, 5 *o'clock*, *P. M.*—Restless, complains of debility; skin soft; pulse 110 per minute; vascularity of the eyes increased; slight hemorrhage from nose and mouth; thirst. Prescription:

| | | |
|---|---|---|
| ℞. | Ext. hyosciam, | grs. xv., |
| | Pulv camphor, | grs. xx., |
| | Muc. g. acacia, | q. s., |
| | M. ft. mass, et divid. in pill., 8. | |

Two every two or three hours, p. r. n., to induce quietude. Continue infus. flor. sambuc. Should hemorrhage increase during the night, give mistur. creasot. half an ounce, every hour or two.

*September* 16*th*, 5 *o'clock*, *A. M.*—Hemorrhage increased immensely; eyes sunken, voice husky; clammy sweat; pulse 120 per minute; blood oozing from the nose, mouth and anus. Notwithstanding the chemical incompatibility of the substances,

℞. Tannin, grs. iij.,
Sul. quinin., grs. ij.,
Tinct. ferri chloridi, gtt. xij., every hour, with a sip of brandy julep; friction to the extremities, with dry capsicum; sinapism to the spine.

5 *o'clock, P. M., same day.*—Hemorrhage partially stopped; pulse fuller, and 100 per minute; skin warm; better expression of countenance; has taken some broth. Ordered the tannin, etc., continued at intervals of two or three hours, according as the sanguineous exudation increased or diminished.

*September 17th.*—Looks cheerful, complains of nothing but weakness; the blood had ceased percolating in the night after a copious discharge of coägula, mixed up with secretions and fecal matter. Let him have porter, and as broth is agreeable to him, let him have that also. He got well.

*Case II.*—Thomas Slater, aged 24 years, stout, but of strumous habit was attacked two days ago; had taken a dose of calomel and jalap which purged him freely; seems despondent; answers questions indifferently; face red; eyes injected; skin somewhat above the natural temperature; capillary circulation sluggish; bowels unmoved since first day of attack, when he took the c. and jal., as above stated. Ordered a mustard pediluvium and pill of mass. hydrarg., ext. col. comp., and ext. hyosciam; infus; flor. sambuc, freely.

*October 3d.*—Bowels moved twice in the latter part of the night; skin warm and moist; pulse 110 per minute; tongue furred and dry; not disposed to answer questions, is dull; at times, partially stupid. Put emp. canthar, to nape of the neck; pillul. hydrarg. gr. iij., every three hours. Continue flor. sambuc. infus.

*October 4th.*—Symptoms same as yesterday; pulse, however, increased in frequency, and complains of "uneasiness" in his bowels; no motion since yesterday. Ordered an enema of an ounce of chloride of sodium dissolved in one pint of infus. lini, and a draught of acet. ammon., with a few drops spts. ether nitros every two or three hours.

*Same day,* 5 *o'clock, P. M.*—Pain in the bowels; nausea, with acid eructations; tongue covered with scarlet blood. Directed hot fomentations to the abdomen, and a spoonful of aqua calcis in some aqua menthæ every hour or two; sinapism to the epigastrium.

*October 5th.*—Nausea abated; bowels easy; skin of dingy appearance; eyes yellow; knitting of the brows; considerable hemorrhage from the mouth and throat. Prescribed: .

℞. Tannin, grs. xviij.,
Sulph. quinin, grs. xij.,
M. ft. pulv., No. 6.

One every two hours with tinct. ferri chloridi, *m*vj., in a wine-glassful of aqua distillat.

*October 6th.*—General appearance somewhat improved; hemorrhage decreasing; a little headache; bowels not moved since yesterday. Let him have an enema of solution of chloride of sodium in flax-seed tea. Continue tannin, quinine, etc.

*October 7th.*—Bowels moved yesterday by injection; no discharge of blood; skin moist, and of normal temperature; tongue clean; complains of debility; pulse 86 per minute; desires food. Directed ale and broth in small quantities; repeat salt enema.

*October 8th.*—Expresses himself much better; desires to sit up; slight perspiration. Continue ale, etc. Convalesced rapidly.

I believe that hemorrhages in yellow fever are salutary, and should not be restrained unless profuse. I was induced to prescribe tannin and tinctferri chloridi in one case when the exudation of blood made its first appearance on the tongue and had reason to regret it; for the hemorrhage ceased entirely after the second dose; the patient got restless; transient delirium ensued; black vomit intervened, and death soon afterwards closed the scene.

I cannot forbear to make a few additional remarks relating to your St. Louis cotemporaries and yellow fever. It seems to me the editors of the St. Louis Journal exhibit a lack of information in regard to the medical literature of New Orleans, or else you would have had no occasion to correct their erroneous expressions about unity of sentiment amongst the doctors of New Orleans in reference to the non-contagiousness, as well as *origin* of yellow fever. Now, if my memory deceives me not, you have a ternary division of the fraternity in your city. When the question of contagion comes up; firstly, you have the unqualified contagionists; secondly, the non-contagion, and thirdly, the *contingent* contagionists; and the only compromise you can make is an agreement to disagree, when in my humble opinion, with such an *authentic record* of *facts* in *regard* to the subject, but one sentiment should possess the minds of all. It is this: That yellow fever, by some contingencies *is* contagious, under other circumstances *not*. This, though not very happily planned, is the "middle ground" for medical men of the present day to occupy, while they adjust their studying cap, and endeavor to investigate the causes of this peculiarity of that singular disease.

In regard to the origin and cause of yellow fever much diversity of opinion also exists in New Orleans and elsewhere, which may be "found on paper." One says yellow fever originates in New Orleans independently of importation, and is caused by a hot sun and *whisky;* another, that it is not indigenous, is introduced from abroad, and requires a certain (unknown) condition of the atmosphere for its propagation and spread. Others, again, agree with the former, so far as relates to its local origin; but one asserts that it is caused by noxious exhalations from the city; another denies it emphatically, and declares that a vitiated atmosphere has nothing to do with it; while another, equally earnest, attributes it to barometrical vicissitudes, or a certain condition indicated by a high dew point; another smiles at the absurdity of all these theories, and affirms that the cause of yellow fever is to be ascribed "to a febritous condition of the atmosphere," and so on; but, "to cap the climax," a new light arises, protesting against all the theories and hypotheses, that was ever promulgated, and avows that *vomito prieto* is of domestic origin in America, and is caused by eating possum! Now, after all these various contradictory and absurd opinions are collected together, analyzed and compared with one another, our judgment irresistibly leads us to the conclusion that the cause of yellow fever cannot be attributed to *terrene emanations,* nor any condition of the atmosphere, whether electric, hydrometric, barometric, nor to any, nor *all* of these conditions combined; nor, I will add, does it bear any relation, whatever, to a redundancy or deficiency of ozone or "marine acid." Well, and furthermore, notwithstanding, in regard to the origin of yellow fever, the mention of Dr. Dowler's, Dr. Hart's, and others' cases occurring in "out of the way places," I am forced to the conviction, "be its work of desolation where'er it may," *yellow fever is neither indigenous to this country, nor a native of any foreign clime.* I assume that it is in the holds of vessels, within the tropics a certain period of time, that are to be found the niduses in which the germs of yellow fever are engendered, developed and perfected, and from them, under favorable circumstances, disseminated on reaching inhabited shores, within certain degrees of latitude.

Few or none will deny that yellow fever has often occurred aboard vessels at sea, under circumstances that forbid the supposition that it could have originated from any other focus of infection than that which existed within the vessels themselves. La Roche has adduced ample and irrefragible testimony to this fact; but by what process of reasoning he arrives at the conclusion that the noxious effluvia generated in the holds of vessels are "identical in nature" with that which is produced on land, I cannot conceive. His language certainly does not involve the inference respecting their propagation from *fomites;* for such an idea he could not entertain for a moment without the grossest inconsistency.

The combination of circumstances under which noxious gases are eliminated from the timbers, bilge-water and cargo of a ship are peculiar; materially different from those upon which depend the exhalations of filthy cities, prison houses, immigrant vessels in temperate latitudes, and paludal districts. Indeed, it is so palpable to the perceptions that it were inconsistent with the plain dictates of common sense to suppose otherwise. Moreover, the peculiar and distinctive characteristics of yellow fever, compared with other fevers, are so different, both as regards its semeiology, and morbid anatomy, that an attempt to identify them were not only supremely absurd, but absolutely humiliating to the understanding of the medical philosopher who would presume to make it. I write *sine invidia;* but am, nevertheless, in earnest. Again, admitting that the fevers of paludal districts are similar to yellow fever, as regards their extrinsic as well as proximate cause, it would be unphilosophical, if not absurd to suppose an identity because "similar causes produce similar effects." "Every thing like is not the same," is an axiom to which every day's experience testifies, and is just as applicable to the phenomena resulting from all scientific investigations, as to the appearances of nature undisturbed.

I have extended this extemporary lucubration further than I intended; yet, nevertheless, would proceed; but forego the pleasure, as I should trespass upon the claims of another paper upon the same subject, designed for future publication.

Very respectfully, yours, etc.,

ROBERT B. S. HARGIS.

